# Application of image analysis for grass tillering determination

**DOI:** 10.1007/s10661-015-4899-2

**Published:** 2015-10-07

**Authors:** Tomasz Głąb, Urszula Sadowska, Andrzej Żabiński

**Affiliations:** Institute of Machinery Exploitation, Ergonomics and Production Processes, University of Agriculture in Krakow, ul. Balicka 116 B, 31-149 Krakow, Poland

**Keywords:** Image analysis, Tillering, Grass, Morphology

## Abstract

Tillering is defined as the process of above-ground shoot production by a single plant. The number of grass tillers is one of the most important parameters in ecology and breeding studies. The number of tillers is usually determined by manually counting the separated shoots from a single plant. Unfortunately, this method is too time-consuming. In this study, a new method for counting grass tillers based on image analysis is presented. The usefulness of the method was evaluated for five grass species, *Phleum pratense*, *Lolium perenne*, *Dactylis glomerata*, *Festuca pratensis* and *Bromus unioloides*. The grass bunches were prepared for analysis by cutting and tip painting. The images obtained were analysed using an automatic procedure with separation of shoots and other objects based on morphological parameters. It was found that image analysis allows for very quick and accurate counting of grass tillers. However, the set of morphological parameters for object recognition must be selected individually for different grass species. This method can be recommended as a replacement for the traditional, time-consuming method in grass breeding.

## Introduction

Variation in grass architecture (e.g. tillering, branching, leafage) profoundly affects light capture, competition and reproductive success and is responsive to environmental factors such as crowding and nutrients limitation. Linking environmental control of branching and tillering with specific modes of gene action might be the key to understanding genes via environmental interactions, and may provide a way to modify crop architecture to achieve greater yields (Doust 2007).

Tillering is defined as the process of above-ground shoot production by a single plant. The number of grass tillers is a characteristic for a particular species and varieties of plants and is usually determined by different environmental factors, such as soil parameters, climate, pathogens and pests (He et al. 2004; Kluse and Diaz 2005; Skalova 2010). Tillering ability determines the effectiveness in preventing soil losses and runoff (Hussein et al. 2007; Xiao et al. 2010).

The number of grass tillers is also an important parameter from the breeders’ point of view. Jeżowski (2008) reported that tillering and bunch diameter in the early stage of growing were the two most important traits influencing the biomass yield of *Miscanthus* clones. Tillering determination helps to identify high-yielding clones or varieties in the search for effective selection of grass hybrids during breeding. Plant tillering is also one of the main parameters which are commonly used in the determination of the stress tolerance of plants (Fernandez and Reynolds 2000; Kotanen and Bergelson 2000; Wachendorf et al. 2001). According to Głowacka et al. (2009), tillering is also a significant factor in judging the resistance of grasses to drought and low temperatures. Zimmermann et al. (2010) reported that reductions in light quality and quantity (also self-shading) were found to suppress the growth, initiation and survival of tillers.

Plant tillering is usually determined by manually counting separated shoots from a single plant. The main disadvantage of this method is that it is highly time-consuming. Calculating the tillering for a single grass plant with more than a hundred tillers usually takes couple of minutes. When the duration and precision of analyses are important, it seems that the application of image processing becomes a reasonable solution. In recent years, image analysis has been widely used for plant recognition, e.g. weed determination in precision agriculture. Selected species determination is based mainly on the characteristic colour and shape of the plant in question (Onyango et al. 2005; Søgaard 2005).

The purpose of this paper was to examine a new method of grass tillering determination based on image analysis and to evaluate the advantages and disadvantages of this method. This method was granted a patent by the Polish Patent Office in 2013 (No. 213091).

## Methods

### Plant material

The field experiment was conducted in Mydlniki near Krakow (50° 04′ N, 19° 51′ E) at the Institute of Machinery Exploitation, Ergonomics, and Production Processes, Agricultural University of Krakow, Poland, in the period from 2006 to 2008.

The seeds of five grass species (timothy-grass *Phleum pratense* L., perennial ryegrass *Lolium perenne* L., cocksfoot grass *Dactylis glomerata* L., meadow fescue *Festuca pratensis* L., prairie grass *Bromus unioloides*) were pre-sown in seed trays and then planted in the experimental field in 2006. A total number of 100 grass bunches, 20 bunches for each species, were prepared. The space between bunches was 0.6 m (in rows and between rows).

### Grass tillering measurements

Measurements were conducted in 2007 and 2008. The tillering was determined during harvesting, three times a year. The tillering of particular grass bunches was determined using two methods: (i) the traditional method by manually counting separated shoots from a single bunch and (ii) image analysis of bunches after cutting. The second method occurred in three main stages: (i) preparing bunches for analysis, (ii) taking photos, and (iii) image analysis.

In the first stage, bunches of grass were cut with a mower, the best was a bar mower with the cutting height of 5 cm. Cut shoots were removed from the observation area.

**Fig. 1:**
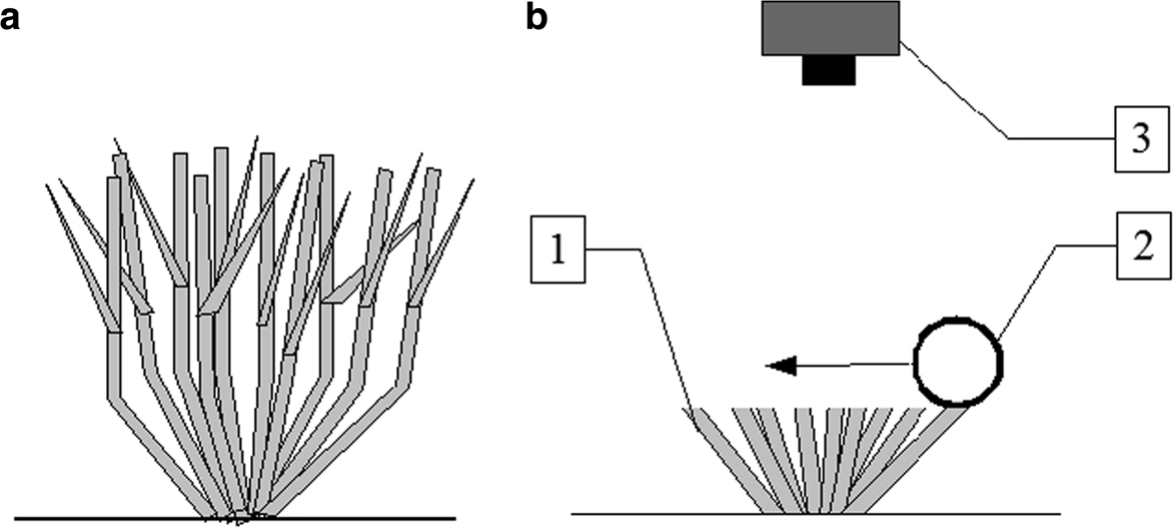
Grass bunch before (**a**) and after (**b**) cutting. *1*—grass stem cut at the 5 cm height, *2*—white painting roller, *3*—digital camera for capturing the image of the bunch

At that stage, the shoot tips were painted with white acrylic paint. The paint was applied with a roller with the diameter of 3–5 cm. The scheme for grass bunches before and after cutting is presented in Fig. 1. Painting shoots white enables sharp, contrasting images of shoot tips to be taken, which helps with the precise separation of objects from their background.

### Image acquisition

In the second stage, the bunches were photographed. In the experiment, the images were collected using a Canon EOS 1000D camera (Canon Inc., Tokyo, Japan) (6.3 effective megapixels) with an EF 50 mm lens. The shutter speed was set at 1/500 s with a sensitivity of 200 ISO, autofocus, and automatic white balance setting. The photographed area was approximately 50 × 50 cm in size, covering the entire bunch of grass. Photos were taken at a distance of about 150 cm, with the camera perpendicular to the soil surface. Pictures were transferred to a computer and saved in JPG format at a resolution of 600 dpi.

### Image processing

**Fig. 2:**
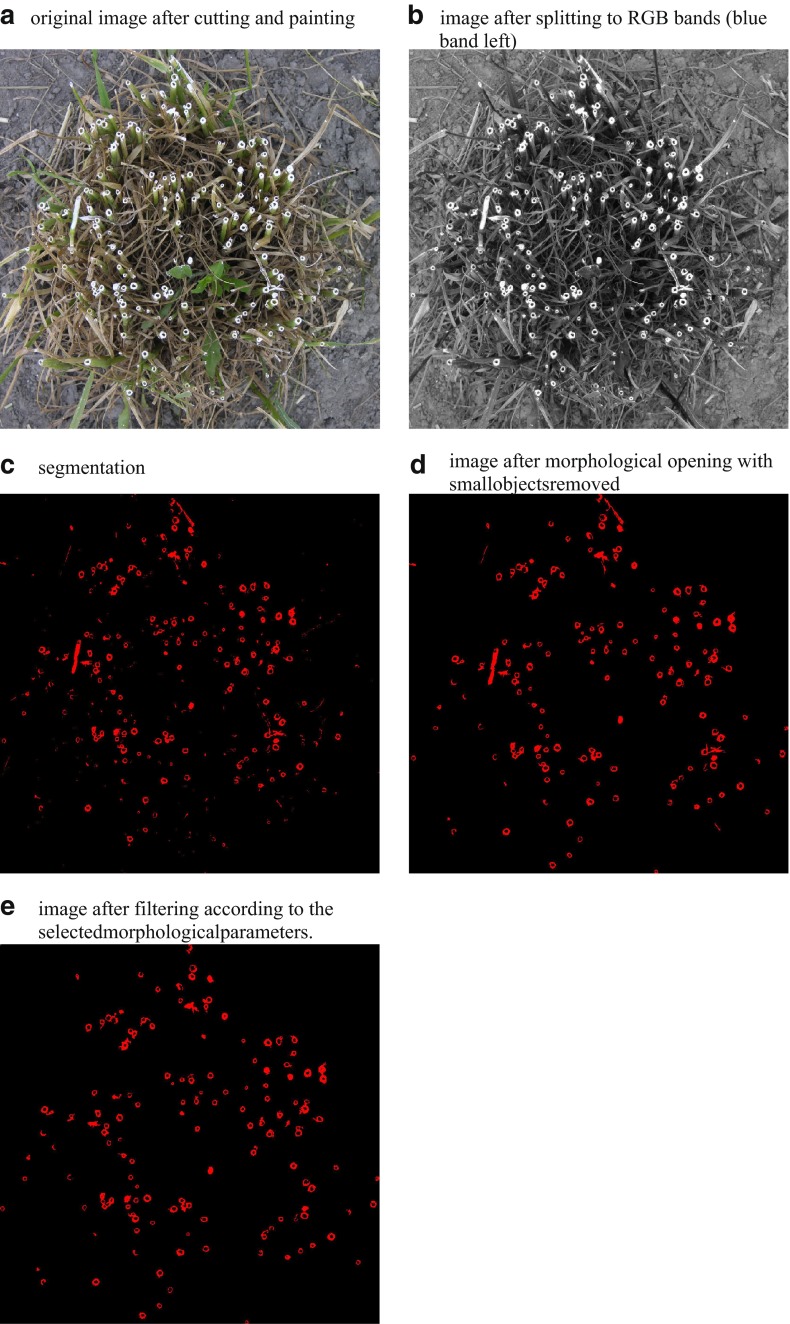
Flow diagram of the algorithm: **a** original image after cutting and painting; **b** image of the grass bunch after splitting to RGB bands (blue band left); **c** segmentation, *red colour*—object of interest, *black colour*—background; **d** image after morphological opening with small objects removed; **e** image after filtering according to the selected morphological parameters
(ii) ImgMaximumContrastThreshold—this operator picks a set of thresholds that give maximal contrast. It automatically selects thresholds that maximize the global average contrast of edges detected by the thresholds across the image (Fig. 2c).
(iii) ImgOpen—this operator performs a morphological opening on all the regions and it is used to remove objects smaller than 200 pixels (Fig. 2d).
(iv) ObjComputeMeasurements—this is an operator which computes a variety of measurements (including shape parameters) for different spatial objects.

The third stage was a computer image analysis. The images were analysed using Aphelion Dev 4.2.0 software for image analysis (ADCIS S.A. and Amerinex Applied Imaging, Herouville Saint-Clair, France). The whole procedure of image analysis was subdivided into four main steps: filtering, segmentation, measurements and object separation (Wojnar and Majorek 1994). The following Aphelion functions were used to prepare this procedure:

(i) ImgColorToRGB—the input image (Fig. 2a) is split into three bands: red (R), green (G) and blue (B). Since they offer the best contrast grey images based on the blue band were prepared for further processing (Fig. 2b).

The following morphometric parameters were calculated:

(i) *A*—area: the area of an object, expressed in pixel^2^
(ii) SF_comp_—compactness: shape factor that is calculated using the following Eq. (1):

1 $$ {SF}_{\mathrm{comp}}=\frac{16\times A}{{P_{\mathrm{Crof}}}^2} $$

(iii) SF_elong_—elongation: the absolute value of the difference between the length of major (*l*_max_) and the minor axes (*l*_min_), divided by the sum of these lengths (Eq. 2). The minor axis is defined as the perpendicular axis to the major axis. This measure is zero for a circle and approaches 1 for a long, narrow ellipse.

2 $$ {SF}_{\mathrm{elong}}=\frac{\left|{\mathrm{l}}_{\max }-{\mathrm{l}}_{\min}\right|}{{\mathrm{l}}_{\max }+{\mathrm{l}}_{\min }} $$

(iv) *A*_conv_—convex area: area of the convex hull (smallest shape that can completely contain an object such that, for any straight line segment connecting two points on the convex hull’s boundary, the entire line segment is contained inside the convex hull) of an object. *A*_conv_ is always greater than or equal to *A*.
(v) *P*_conv_—convex perimeter: perimeter of the convex hull of an object and expressed in calibrated units.
(vi) SF_conv_—convexity: This is a measurement of the particle edge roughness and it is equal to the area of an object divided by the area of its convex hull, following Eq. (3):

3 $$ {SF}_{\mathrm{conv}}=\frac{A}{A_{\mathrm{conv}}} $$

(vii) CMA—convex minimum angle: the minimum of the angles formed by the adjacent pairs of line segments that comprise the polygonal boundary of an object, given in radians.
(viii) SMD—symmetry mean difference: mean of the absolute values of the length difference between the centroid (the arithmetic mean position of all the points in the shape) and two opposite boundary points of an object (*l*_*A*_ and *l*_*A*′_). With *N* equal to the number of boundary points divided by two, the measurement’s formula is (Eq. 4):

4 $$ SMD=\sum_{i=0}^{N-1}\left|{\mathrm{l}}_{A(i)}-{\mathrm{l}}_{A^{\hbox{'}}(i)}\right| $$

(ix) FD_max_—maximum Feret diameter in an object’s set of Feret diameters
(x) FD_min_—minimum Feret diameter in an object’s set of Feret diameters
(xi) SF_sfer_—Feret Pentland sphericity: shape factor introduced by Pentland (1927) (Eq. 5):

5 $$ {SF}_{\mathrm{sfer}}=\frac{4\times A}{\pi \times {FD_{\max}}^2} $$

The calculated morphometric parameters cover the object’s dimensional features, such as area (*A*, *A*_conv_), perimeter (*P*_conv_, CMA) and diameter (FD_max_, FD_min_), expressed in calibrated units, as well as dimensionless shape factors (SF_comp_, SF_elong_, SF_conv_, SMD, SF_sfer_).

### Statistics

The basic statistics (number of measurements, mean, minimum and maximum values, and standard deviation) for data obtained by the two tested methods of tillering determination are presented in Table 1. Data were analysed using the statistical package Statistica v. 10.0 (StatSoft Inc., Tulsa, OK, USA).

**Table 1:** Results of tillering determined by manual counting (*O*) and image analysis of grass bunches (*E*)

	*Phleum pratense*	*Dactylis glomerata*	*Festuca pratensis*	*Bromus unioloides*	*Lolium perenne*
*n*	100	96	92	100	107
*O*	136.2	123.4	129.8	132.6	139.9
SD	68.1	65.1	69.8	64.6	60.2
*O*_min_	27	18	32	15	32
*O*_max_	272	264	329	244	356
*E*	143.5	149.1	138.0	141.3	151.2
SD	53.7	52.8	58.1	53.1	52.1
*E*_min_	42	26	26	10	35
*E*_max_	265	258	243	228	242

*n* number of observations, *O* number of tillers determined by manual counting, *SD* standard deviation, *O*_*min*_ minimal value of *O*, *O*_*max*_ maximal value of *O*, *E* number of tillers determined by image analysis, *E*_*min*_ minimal value of *E*, *E*_*max*_ maximal value of *E*

Linear discriminant analysis (LDA) was used to classify the different objects based on their morphometric parameters. LDA is a technique commonly used to classify unknown groups characterized by quantitative and qualitative variables. A training set of objects used for parameter selection (approximately 1200 objects, 20 % of total number of objects) was divided into two classes: *shoots* and *non-shoots*. The parameters were selected on the basis of three statistical variables: tolerance, F-to-remove and Wilks’ lambda. The tolerance value indicates the proportion of a variable’s variance not accounted for by other independent variables in the model. F-to-remove values define the power of each variable in the model, and these are useful to describe what happens if a variable is removed from the current model. The values of the Wilks’ Lambda show the discriminatory ability of the LDA function.

The collinearity between the independent variables poses a statistical problem in regression models. The common diagnostic test for this is the variance inflation factor (VIF) (Zuur et al. 2010). Following the suggestion of Dormann et al. (2013), the VIF was computed and applied in a stepwise deletion of parameters according to decreasing VIF values, until a threshold of 10 was reached.

Regression models were determined when correlation coefficients were significant. The linear regression slope and intercept were tested for significant differences from 1 and 0, respectively, using an *F* test. The root mean square error (RMSE), mean absolute error (MAE), mean bias error (MBE) and coefficient of determination (*R*^2^) were used to evaluate the accuracy of the implemented models and calculated as Eqs. 6, 7, 8 and 9:

6 $$ \mathrm{RMSE}=\sqrt{\frac{\sum_{i=1}^n{\left({E}_i-{O}_i\right)}^2}{n}} $$

where *O*_*i*_ is the manually counted grass tillering, *E*_*i*_ is the tillering estimated by image analysis, and *n* is the number of test data.

7 $$ MAE=\frac{\sum_{i=1}^n\left|{E}_i-{O}_i\right|}{n} $$

8 $$ MBA=\frac{\sum_{i=1}^n{\left({E}_i-{O}_i\right)}^2}{n} $$

9 $$ {R}^2=1-\frac{\sum_{i-1}^n{\left({O}_i-{E}_i\right)}^2}{\sum_{i=1}^n{\left({O}_i-\overset{-}{O}\right)}^2} $$

## Results and discussion

The first problem, encountered during the procedure, was to distinguish the painted shoot tips from the background. The main components of the background are soil and grass leaves (living and thatch). Spectral analyses of soil show that all three bands (red, green and blue) are presented in equal proportions. It is a characteristic for soil that it has considerably fewer peak and valley variations in the reflectance (Langner et al. 2006). However, the reflectance of soil can vary depending on water and organic matter content. Picture quality is also affected by changes in illumination, which occur frequently due to changing weather, especially on partly cloudy days. A similar problem was also reported by Burgos-Artizzu et al. (2011). They recommended taking the pictures on cloudy days when the light is diffused and the contrast is lower. Plants show relatively low values in the red and the blue regions of the visible spectrum, with a peak in the green spectral band (Perez et al. 2000). Some colour indices based on the red, green and blue bands have been proposed to distinguish green leaves from soil as a background. Woebbecke et al. (1995) found that the excess green vegetation index 2G-R-B works well in the segmentation of vegetation against background. Meyer and Neto (2008) improved this index. They proposed an index based on excess green and red, which worked especially well for fresh wheat straw backgrounds. The normalized difference vegetation index (NDVI) recommended by Perez et al. (2000) uses only green and red channels (G-R)/(G + R). In this research, the blue band was chosen for further investigation according to Wiles (2011). This method was found to be sufficient to distinguish the region of interest. In this study, it was not necessary to use indices that are more complex. The separated tips of shoots were painted white, and it was much easier to distinguish them from the background than it would have been to separate all the green plants from the soil.

The next step in the procedure, after band splitting, was segmentation. The accuracy of segmentation depended on the difference between the objects (shoot tips) and their surroundings. In the image analysis of plant material, both automatic and manual segmentation are used. Automatic segmentation techniques have significantly developed in recent years (Sofou et al. 2001; Sezgin and Sankur 2004). Szala (2001) stated that, from the segmentation point of view, the best grey image should have two sets of pixels significantly different in grey level values. This was also confirmed by Wojnar et al. (2002) who indicated that automatic segmentation could be recommended for two maximum histograms, with clearly separated peaks. In this research, it was possible to use automatic segmentation (maximum contrast method) based on the histogram of the image. This method has also been used in weed detection (Philipp and Rath 2002). The result of the image analysis procedure was a set of objects. However, these objects presented not only the tips of grass shoots but also fragments of leaves and other debris. So, the next step in the image analysis procedure was filtration using morphological features of these objects.

Seven object parameters (out of 11) remained after performing the VIF stepwise method for deleting multi-collinear variables (Table 2). Grass species differ slightly in terms of the number of parameters which are included in LDA models (Table 3). All object parameters were used for recognition shoots of *F. pratensis* and *L. perenne*. Shoots of *D. glomerata* need six parameters, and for other species, five object parameters were included in the LDA model. Five parameters, namely CMA, SF_circ_, SF_conv_, SF_elong_ and SMD, were found to be very useful for all the investigated grass species. SF_circ_ had the best prediction value for *D. glomerata* and *B. unioloides* according to F-to-remove and Wilks’ lambda. SF_elong_ had the most discriminant value for *F. pratensis* and *L. perenne.* The rest of the 11 proposed morphological parameters were found to be useless in the procedure of grass shoot tip recognition in bunch images.

**Table 2:** Variance inflation factors (VIF) for the morphological object parameters as explanatory variables in linear discriminant analysis (LDA)

	*Phleum pratense*	*Dactylis glomerata*	*Festuca pratensis*	*Bromus unioloides*	*Lolium perenne*
*A*	32.96	35.79	9.89	48.53	9.06
*A*_conv_	23.37	48.11	11.91	38.52	32.31
CMA	1.36	1.56	1.68	1.89	1.37
FD_max_	382.04	365.59	566.91	246.34	746.15
FD_min_	44.81	29.09	27.91	28.43	72.19
*P*_conv_	493.86	560.46	716.72	357.48	1036.19
SF_comp_	4.15	2.63	3.48	2.76	4.03
SF_conv_	5.12	4.75	8.93	5.43	4.76
SF_elong_	7.74	4.67	3.37	6.67	5.71
SF_sfer_	13.12	7.59	5.96	10.43	6.94
SMD	7.70	5.46	7.76	8.28	9.19

*A* area, *A*_*conv*_ convex area, *CMA* convex minimum angle, *FD*_*max*_ maximum Feret diameter, *FD*_*min*_ minimum Feret diameter, *P*_*conv*_ convex perimeter, *SF*_*comp*_ compactness, *SF*_*conv*_ convexity, *SF*_*elong*_ elongation, *SF*_*sfer*_ Feret Pentland sphericity, *SMD* symmetry mean difference

**Table 3:** The selected object morphological features for grass shoot recognizing using linear discriminant analysis

	*Phleum pratense*			*Dactylis glomerata*			*Festuca pratensis*			*Bromus unioloides*			*Lolium perenne*		
	*T*	*F*	*Λ*	*T*	*F*	*Λ*	*T*	*F*	*Λ*	*T*	*F*	*Λ*	*T*	*F*	*Λ*
*A*							0.101	4.752	0.139				0.110	1.932	0.100
CMA	0.733	2.115	0.119	0.640	0.264	0.216	0.596	4.274	0.138	0.529	0.482	0.182	0.729	1.415	0.100
SF_comp_	0.241	6.239	0.123	0.381	36.876	0.278	0.288	0.081	0.133	0.362	0.630	0.182	0.248	10.326	0.107
SF_conv_	0.195	0.431	0.118	0.210	6.022	0.226	0.112	0.093	0.133	0.184	0.444	0.182	0.210	1.049	0.100
SF_elong_	0.129	15.140	0.131	0.214	0.496	0.216	0.297	23.583	0.162	0.150	0.186	0.181	0.175	24.488	0.119
SF_sfer_				0.132	12.894	0.238	0.168	23.599	0.162				0.144	0.038	0.099
SMD	0.130	0.053	0.118	0.183	5.269	0.225	0.129	0.238	0.133	0.121	0.202	0.181	0.109	2.865	0.101

*T* tolerance, *F* F-to-remove, *Λ* Wilks’ lambda, *A* area, *CMA* convex minimum angle, *SF*_*comp*_ compactness, *SF*_*conv*_ convexity, *SF*_*elong*_ elongation, *SF*_*sfer*_ Feret Pentland sphericity, *SMD* symmetry mean difference

The results present a good separation for shoot tips from the bunch images of all tested grass species. However, individual species need different sets of object morphological parameters. This can be ascribed to differences between tested grass species in terms of their shoot and leaf morphology. The investigated grass species differed in their vegetative stem shape and size. Both *L. perenne* and *F. pratensis* had round, thin stems with diameters up to 2 mm. The stem diameter for *P. pratense*, *D. glomerata* and *B. unioloides* was above 2 mm. The stem section shapes of *P. pratense* and *B. unioloides* were round, whereas the *D. glomerata* stem had a flattened base, which distinguished it from the other grasses. It was expected that for this species with flattened stems, there would be a problem with stem detection on the image and distinguishing them from cut leaves. However, this was not a significant problem, probably due to the small number of leaves at the cutting height. *D. glomerata* is a tall grass with a height up to 1.5 m, and in the lower part of the plants, there is low leafage. On the image obtained, there were not numerous leaf sections that could impede the analysis.

For image analysis of *L. perenne*, it was necessary to use more morphological parameters in comparison to other tested grasses. This species has dense leafage with numerous leaves on the lower part of the plant. The leaves are usually thin, with an area close to the diameter of the stem. The difficulty in counting stems using image analysis is connected with the problem of distinguishing stem sections and leaves on the images of bunches. *P. pratense*, with a round and thick stem and lower leafage on the lower parts of stem, needed only four morphological parameters to recognize the stem sections.

The parameters of linear regression for the relationships between the results for the number of grass tillers obtained by the two abovementioned methods are presented in Table 4. The results showed that all investigated grass species had a similar precision of tiller determination using the method based on image analysis. The value of the coefficients of determination, *R*^2^, for the linear regression between the tested methods was 0.858 on average. The error indices for the linear regression, such as RMSA, MAE and MBA, also showed a high level of accuracy.

**Table 4:** Parameters and accuracy indexes of the linear regression models for the relationships of two methods of the grass tillers counting. The correlation coefficient (*r*), root mean square error (RMSE), mean absolute error (MAE), mean bias error (MBE), coefficient of determination (*R* ^2^)

Statistics	*Phleum pratense*	*Dactylis glomerata*	*Festuca pratensis*	*Bromus unioloides*	*Lolium perenne*
*r*	0.945*	0.895*	0.897*	0.932*	0.892*
Regression parameters					
Slope	1.13	1.21	1.11	1.12	1.02
*P* value	<0.0001	<0.0001	<0.0001	<0.0001	<0.0001
Intercept	−14.2	−11.5	−29.8	−12.4	−22.1
*P* value	<0.0001	<0.0001	<0.0001	<0.0001	<0.0001
Accuracy measures					
*RMSE*	17.2	25.1	22.1	19.4	33.1
*MAE*	20.7	20.2	25.6	18.4	28.4
*MBA*	394.3	821.5	795.4	321.5	6.95.1
*R* ^2^	0.902	0.854	0.812	0.903	0.821

*r* correlation coefficient, *RMSE* mean square error, *MAE* mean absolute error, *MBE* mean bias error, *R* ^*2*^ coefficient of determination

*Significant at *P* > 0.05

The main advantage of the described method is its speed of use. Preparing the bunch (painting) and taking the picture usually take several seconds per bunch, whereas counting stems in a bunch in the traditional method takes up to 5 min. In experiments with hundreds of bunches, this difference becomes very significant. However, it should be underlined that this method can be recommended only in breeding trials where separate bunches are cultivated and not in field trials with pasture mixtures and without weeds.

## Conclusions

The use of image analysis enables very quick and accurate counting of grass tillers. This method could be recommended to replace the traditional, time-consuming method in grass breeding. The grass bunches should be sown separately and they must be prepared for analysis by cutting and tip painting. The images obtained can be analysed using an automatic procedure. Separation of shoot tips and other objects is based on morphological parameters. However, individual species need different sets of object morphological parameters which can be ascribed to differences in their shoot and leaf morphology. Further investigation is recommended in order to select the individual sets of morphological parameters for other grass species.
